# Molecular Dermatopathology in Malignant Melanoma

**DOI:** 10.1155/2012/684032

**Published:** 2011-10-20

**Authors:** Marie-Annick Reginster, Claudine Pierard-Franchimont, Gérald E. Piérard, Pascale Quatresooz

**Affiliations:** Department of Dermatopathology, University Hospital Sart Tilman, 4000 Liège, Belgium

## Abstract

At present, immunohistochemistry is taken for granted in the establishment of malignant melanoma (MM) diagnosis. In recent years, molecular diagnosis in dermatopathology has benefited from a vast array of advances in the fields of genomics and proteomics. Sensitive techniques are available for detecting specific DNA and RNA sequences by molecular hybridization. This paper intends to update methods of molecular cytogenetics available as diagnostic adjuncts in the field of MM. Cytogenetics has highlighted the pathogenesis of atypical melanocytic neoplasms with emphasis on the activation of the mitogen-activated protein kinase (MAPK) signalling pathway during the initiation step of the neoplasms. 20 to 40% of MM families have mutations in the tumour suppressor gene p16 or CDKN2A. In addition, somatic mutations in p16, p53, BRAF, and cKIT are present in MM. Genome-wide scan analyses on MM indicate positive associations for genes involved in melanocytic naevi, but MM is likely caused by a variety of common low-penetrance genes. Molecular dermatopathology is expanding, and its use in the assessment of melanocytic neoplasms appears to be promising in the fields of research and diagnosis. Molecular dermatopathology will probably make its way to an increased number of diagnostic laboratories. The expected benefit should improve the patient management. This evolution points to a need for evolution in the training requirements and role of dermatopathologists.

## 1. Introduction

In some instances, identifying cutaneous malignant melanoma (MM) in routine histopathology may prove to be a challenging exercise [1–3]. The diagnostic process is hampered by a series of clinical and histopathological limitations in both the definition of objective criteria and establishment of undisputable diagnostic consensus. Over the past decades, the development of immunopathology notably shifted the diagnostic procedure from descriptive morphology to molecular histopathology [4–8]. Immunopathology helps distinguishing some atypical melanocytic neoplasms and supports refined MM staging [9–18]. In recent years, other progresses were made beyond regular microscopy in the fields of molecular biology and molecular morphology for the detection of pathogenic mutations expressed in DNA, RNA, and proteins. Such sensitive procedures applied to MM help in the diagnosis, classification, and outcome prediction, as well as in selecting and monitoring therapy [19–24]. Of note, mutations altering a protein due to amino acid substitution have to be distinguished from mutations occurring in a given amino acid and corresponding to silent mutations or polymorphism.

The aim of this paper was to revisit recent insights in molecular dermatopathology shedding some light on the pathogenesis and diagnosis of MM. The accent will be on molecular cytogenetics referring to the molecular structure and the function of chromosomes. The covered techniques are currently used for research or diagnostic purposes.

## 2. Complementary Sampling Methods

Two special sampling methods are particularly suited for some methods used in molecular dermatopathology.

Laser cutting dissection helps isolating circumscribed cell populations under the microscope in an attempt at increasing homogeneity of isolates based on morphological criteria [25, 26].

Tissue microarrays correspond to an ordered set of minute tissue cores (roughly 0.6 mm in diameter) obtained from a variety of specimens and embedded in a single paraffin block. For comparative purposes, immunohistochemistry and a range of downstream molecular methods are conveniently applied to tissue microarrays [18, 27]. For instance, tissue microarrays from different MM samples at different stages of progression were used to disclose distinct molecular alterations [27]. In this field, immunohistochemistry on tissue microarrays has identified osteopontin expression as the first feature acquired during the initial step of MM invasion [27].

## 3. Molecular Morphology and Biology at a Glance

### 3.1. In Situ Hybridization

In situ hybridization (ISH) provides morphological demonstration of specific DNA and RNA sequences in tissue sections, single cells, or chromosome spreads. ISH relies on labelled single- or double-strand DNA or RNA sequences containing complementary sequences that hybridize to cellular DNA or RNA under appropriate conditions to form stable hybrids. The optimal probe length for ISH results in maximized tissue penetration and high hybridization rate. It averages 50–300 bases.

Primed in situ (PRINS) labelling relies on a primer-mediated DNA synthesis carried out in situ on tissue sections. The technique starts with annealing an oligonucleotide DNA primer adjacent to the DNA region of interest. This molecular structure serves as a primer for the Tag polymerase incorporating the four nucleotides dATP, dGTP, dCTP, and the labelled dUTP which is conveniently revealed using immunohistochemistry. These ISH and PRINS methods are used for localizing DNA sequences and specific mRNA in cells and tissues, as well as visualizing chromosomes allowing interphase cytogenetics and detection of specific mRNA. Thus, HIS and PRINS methods were developed using chromosome-specific probes or oligonucleotides for the detection of numerical and structural chromosome aberrations in interphase nuclei in both fresh and formalin-fixed tissue sections.

### 3.2. Chromosomal G Banding Method

Chromosomal G banding (CGB) detects gross aberrations in chromosomes under microscopic examination [18, 28]. Cell cultures from fresh MM sampling are followed by stabilization of mitotic figures in metaphase. Enzyme digestion followed by histochemical staining reveals CGB. Of note, subtle chromosomal aberrations including point mutations, small insertions, and deletions, as well as discrete rearrangements are not disclosed using the CGB method [24].

### 3.3. FISH Method

Fluorescence in situ hybridization (FISH) relies on hybridization of fluorescent complementary DNA probes recognizing specific genes or DNA oligonucleotides [29]. The method is performed on fresh or formalin-fixed tissue sections, as well as on nuclei spreads and DNA microarrays. The number of spots per nucleus is indicative of the copy number of the scrutinized chromosome locus. Sequences of the whole genome, centromeres, telomeres, and specific gene regions are conveniently used as probes. A translocation is detected by a spot splitting into two parts, or by different fluorescent probes that hybridize on each of the translocated genes. 

FISH helps identifying mutated cells including those in the apparently normal skin at distance from the MM [30]. Recent FISH variants were developed in various ways [24, 31]. FISH allows detecting subtle chromosome aberrations in MM including four targeted genes, namely, CEP6, RREB1, MYB and CCND1 [32–35].

One pitfall of the FISH method results from the partial entrapping of the nuclei volume in the tissue sections causing technical omission of chromosome segments. Hence, a number of nuclei must be scrutinized for ensuring a reliable assessment of the copy number of the probe [36].

### 3.4. CGH Method

Comparative genomic hybridization (CGH) compares DNA from a melanocytic neoplasm to DNA from a normal reference tissue of the same patient. The CGH method is conveniently performed using fresh or formalin-fixed tissue. The DNA labelled with different fluorochromes is subsequently hybridized on normal metaphase chromosomes or on arrays of small spots of DNA [24]. Data are expressed as a gain or loss of copy number [37]. CGH has identified some genome aberrations and imbalance in MM [38], but only when genome aberrations are enough represented in neoplastic cells [31]. CGH is mainly used in research settings [38, 39] where it helps identifying genomic signatures distinguishing different MM types [40–42]. Of note, Spitz naevi (melanocytomas) [43] contain genetic aberrations corresponding to single 11 p gains [19, 38]. MM arising in congenital melanocytic naevi shows CGH patterns comparable to regular MM, while atypical nodules (melanocytomas) contain numerical aberrations of entire chromosomes, which are seen only in a minority of MM [44].

### 3.5. Gene Microarray Method

Gene microarrays also named DNA chips allow scrutinizing RNA and microRNA (miRNA) expression, as well as detecting DNA mutations and polymorphism [45–47]. The method is applicable to fresh or formalin-fixed tissue [47]. The procedure involves immobilization of specific DNA sequences on a solid platform, to which complementary labelled DNA hybridizes. The labelled DNA is obtained from reverse transcription of mRNA extracted from the test tissue. The immobilized DNA corresponds to either oligonucleotides or complementary DNA [24]. Following the hybridization process, the labelled spots are scrutinized. The complexity of the multiple data requires adequate statistical analysis and computerized mathematical models.

Gene microarray signatures were offered for molecular classification of MM and naevi in diagnostic dermatopathology [47]. In such screening, 36 different gene signatures were found between melanocytic naevi and MM.

### 3.6. PCR and RTP-CR Methods

The PCR (polymerase chain reaction) substrate corresponds to DNA or RNA (reverse transcriptase, RT-PCR), extracted from fresh or formalin-fixed tissue. The method corresponds to a chemical reaction driven by cyclic changes in temperature leading to a specific and exponential amplification of small fragments of the target DNA or RNA over a short period of time. PCR was applied to the identification of MM micrometastases [48] using selected markers including me20m, PLAB, SPP1, CAPG, and CTSB [24, 26, 49]. 

While immunohistochemistry improves the sensitivity of metastasis detection by 10–45% compared with regular histopathology, RT-PCR for MM-related marker gene expression, like tyrosinase and Melan A-Mart-1, was reported to increase the detection of suspected occult metastases up to 70% [24]. Indeed, RT-PCR is expected to detect one MM cell out of 10^6^-10^7^ non-MM cells, while immunohistochemistry probably detects one MM cell in about 10^4^-10^5^ non-MM cells [21]. The relevance of PCR detection of MM micrometastases in lymph nodes was, however, challenged because of the apparent lack of prognostic value of the findings [50]. Indeed, in addition to the identification of MM micrometastasis within sentinel lymph nodes, the size and location of the metastasis should be assessed [51]. Thus, the role of nonmicroscopic methods to investigate the sentinel lymph node remains doubtful. In such procedure, a histopathological assessment of the location, size, and nature of the suspected MM cells is lacking and misinterpretation of nodal nevus cells as metastasis may occur. This drawback might be overcome by using highly specific methods detecting only MM cells [52], but the size and location of the micrometastases remain unassessed.

In situ PCR-based amplification of the target nucleic acid sequences may be performed before performing ISH. In situ PCR is performed on cells or tissues are fixed and permeabilized respecting the morphology and allowing access of the PCR probes to the intranuclear sequences that have to be amplified. Cell suspensions or tissue sections are overlaid with PCR reagents, sealed off with a coverslip, and submitted to thermal cycling. The PCR products are detected using PCR probes (indirect in situ PCR) or through direct detection of labelled nucleotides incorporated into PCR products (direct in situ PCR).

### 3.7. MLPA Method

 Multiplex ligation-dependent probe amplification (MLPA) is based on annealing up to 45 probes each consisting of two oligonucleotides that hybridize next to each other on a specific chromosome region [53]. In addition to target-specific sites, both oligonucleotides, contain a universal PCR primer. In addition, one oligonucleotide contains a so-called stuffer sequence showing a probe-specific length. After ligation of the hybridized adjacent oligonucleotides the probe is amplified using PCR primers. As each probe has a unique length due to the stuffer sequence, electrophoresis conveniently separates and quantifies the amount of PCR product indicating the DNA copy number [24]. Mutation-specific MLPA combines copy number detection and hot-spot mutations in a single assessment [54].

### 3.8. HRMA Method

High-resolution melting analysis (HRMA) is based on the DNA dissociation after heating at high resolution. This procedure allows for the detection of single-base pair sequence variations [55]. This rapid method detects hot-spot mutations and possibly identifies mutated cells when present in at least 5% of a background of cells showing wild-type DNA [56].

## 4. Germline and Somatic MM Mutations

Multiple naevi associated with increased MM risk predominantly occur in families with p16 or CDKN2A mutations [57]. However, searching for p16 or CDKN2A mutations is not clinically relevant in other MM patients as the probability of detecting a mutation is small [58]. The prevalence of familial p16 mutations is related to the number of MMs in relatives [57]. More than 30 different p16 mutations have been reported so far [58]. Globally, p16 mutations are present in 25–40% of MM families with more than 2 MMs, while the proportion of p16 mutations is lower in families with 2 MMs only [59]. In addition, individuals with multiple primary MM are more likely to have p16 mutations [60]. 

The p16 gene produces two different polypeptides, namely, the p16/INK4a and the p14/ARF proteins. Both of them participate in the control of the cell cycle of proliferation. According to the exon mutation, the p16 and/or p14 functions are altered. Indeed, most MMs are unrelated to germline p16 mutations especially in the nonfamilial MM. Many other common MM genes of lower penetrance are probably involved in MM. These mutations increase with the progression of the disease [61]. Multiple dysplastic naevi are a marker of MM risk and susceptibility to other cancers as well [62]. MM is therefore likely to share cancer genes with other neoplasms because MM is common in the family cancer syndromes. Individuals with BRCA2 and BRCA1 mutations have an increased risk of MM on the skin and eyes [63].

Genetic aberrations in cutaneous MM correlate with specific phenotypes [64]. Of note, the progression from a melanocyte to a MM cell is supported by a series of morphological changes, and a series of currently unravelled genetic aberrations. Both the mitogen-activated protein kinase (MAPK) signalling pathway and the PTEN/AKT pathway are involved in the growth control of melanocytes [65]. Activation of these pathways following somatic mutations in the RAS and RAF genes might represent one of the initial steps in the development of melanocytic naevi [66]. The BRAF oncogene on chromosome 7Q34 is commonly mutated in over 70% of MMs [67], but this mutation is absent in giant congenital melanocytic naevi [68]. MM with multiple naevi and BRAF mutation occur more frequently at a younger age and on intermittently sun-exposed sites [69]. By contrast, p53 mutations are more common in lentigo maligna or MM developed on chronically sun-exposed sites of older patients showing prominent actinic damage [70]. Distinct somatic gene mutations such as at the cKIT locus were identified in other MMs, particularly on the mucosa, palms, and soles [71], and in hyperpigmented MM [15]. Genes potentially involved in MM metastases include those involved in the MAPK pathway, such as BRAF and RAS. New MM therapies targeting the BRAF and cKIT pathways call for genotyping MM in order to select the proper patients [71–76].

Blue naevi, Spitz melanocytomas, congenital melanocytic naevi, and uveal MM do not or rarely contain BRAF mutations [68]. By contrast, they contain other mutations such as in the NRAS or HRAS genes [77]. In addition, somatic mutations of GNAQ in the RAS-like domain were found in both uveal MM and blue naevi [78]. Similarly to BRAF mutations, the RAS and GNAQ mutations may cause MAPK activation and form an alternative route for melanocytic neoplasia [55]. Even if all these mutations seem to represent early events in the development of melanocytic neoplasms, they do not cause melanocytic progression towards MM.

## 5. Conclusion

Most of the molecular methods available in dermatopathology currently remain research-based approaches. In such setting, molecular diagnosis appears increasingly important for predicting the biological behaviour of MM. New markers correlating with poor prognosis have been reported. It appears that MM is a genetically heterogeneous neoplasm with different risk phenotypes and genotypes.
